# Cognitive Impairment in Chronic Kidney Disease Across Different Stages: The Role of Structural and Perfusion‐Driven Functional Connectivity Changes

**DOI:** 10.1002/brb3.70330

**Published:** 2025-02-17

**Authors:** Xiaoyan Bai, Lijun Song, Xu Liu, Wenbo Yang, Mingan Li, Boyan Xu, Zhenghan Yang, Zhen‐Chang Wang, Hao Wang

**Affiliations:** ^1^ Department of Radiology Beijing Friendship Hospital, Capital Medical University Beijing China; ^2^ Department of Nephrology Beijing Friendship Hospital, Capital Medical University Beijing China; ^3^ MR Research GE Healthcare Beijing China

**Keywords:** cerebral blood flow, chronic kidney disease, cognitive impairment, functional connectivity, gray matter volume

## Abstract

**Introduction:**

Chronic kidney disease (CKD) is associated with cognitive impairment (CI), yet the exact pathophysiological mechanisms remain unclear. This study aims to investigate the alterations in gray matter volume (GMV) and cerebral blood flow (CBF) across CKD stages, identify co‐changed brain regions, explore abnormal seed‐based functional connectivity (FC) in patients with CKD, and investigate the correlation between the abnormal brain regions and neuropsychological test scores.

**Methods:**

Two hundred and eight participants (66 healthy controls, 70 CKD Stages 1–3a, and 72 CKD Stages 3b–5) were consecutively recruited and underwent high‐resolution T1‐weighted imaging, arterial spin labeling, and functional MR imaging. The imaging parameters were compared among three groups, and correlations with MoCA scores were analyzed.

**Results:**

Compared to CKD 1–3a group, the bilateral fusiform gyrus (FFG.L and FFG.R) exhibited reduced GMV, increased CBF, and decreased FFG.L‐FC with bilateral inferior frontal gyrus, triangular part (IFGtriang.L and IFGtriang.R), left middle occipital gyrus (MOG.L), and left hippocampus (HIP.L), as well as decreased FFG.R‐FC with bilateral median cingulate and paracingulate gyri (DCG.L and DCG.R), left superior frontal gyrus, medial (SFGmed.L), IFGtriang.L, and right middle temporal gyrus (MTG.R) in CKD 3b–5 group. A negative correlation was observed between the MoCA scores and FFG.L‐FC with right middle frontal gyrus (MFG.R), IFGtriang.L, IFGtriang.R, HIP.L, and left putamen in patients with CKD 1–3a.

**Conclusion:**

Brain structural and perfusion alterations may underlie the reduced FC between fusiform gyrus and cognitive‐related regions, providing potential neuroimaging evidence for the neuropathological mechanisms of CI in patients with different stages CKD.

## Graphical abstract

Patients with CKD, particularly in early stages, may exhibit changes in GMV and CBF, which can lead to decreased FC between the fusiform gyrus and cognitive‐related regions in advanced‐stage CKD. CKD, chronic kidney disease; CKD3b‐5, patients with stage 3b‐5 chronic kidney disease; CKD1‐3a, patients with stage 1‐3a chronic kidney disease; HC, healthy control; MNI, montreal neurological institute; FC, functional connectivity; GRF, Gaussian random field. FFG.L, left fusiform gyrus; FFG.R, right fusiform gyrus.

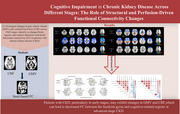

## 1 Introduction

In recent decades, the incidence of chronic kidney disease (CKD) has markedly escalated, affecting over 10% of the global population (Levey and Coresh 2012; Kovesdy 2022). CKD is characterized by abnormalities in kidney structure or function that persist for more than 3 months with health implications (Levey and Coresh 2012). On the basis of the estimated glomerular filtration rate (eGFR), the condition is classified into five stages, ranging Stage 1–5 (CKD Stages 1–5, eGFR 15 mL/min per 1.73 m^2^ to < 60 mL/min per 1.73 m^2^). These stages are often grouped into early‐stage CKD (Stages 1–3a, eGFR ≥ 45 mL/min per 1.73 m^2^) and advanced‐stage CKD (CKD Stages 3b–5, eGFR < 45 mL/min per 1.73 m^2^), in accordance with previous classification standards (Stevens et al. 2013; Hu et al. 2018). Patients with CKD often exhibit cognitive impairment (CI), which manifests as a decline in orientation and attention, concept formation and reasoning, language, memory, and executive functions with poor life quality and overall prognosis (Murtaza and Dasgupta 2021; Berger et al. 2016). Notably, numerous studies have highlighted the severity of CI is closely linked to the CKD stage (Pépin et al. 2022; H. Wang et al. 2023; Scheppach et al. 2023). However, the pathophysiological mechanisms underlying cognitive dysfunction in CKD remain poorly understood. A prevailing view suggests that uremic neurotoxins interacting with neural progenitor cells, the brain vasculature, and monoaminergic neurons may contribute to the pathogenesis of CKD‐related CI (Viggiano et al. 2020). These interactions may result in abnormal brain changes, including cerebral atrophic, abnormal cerebral perfusion, and alterations in brain activity, which ultimately affect cognitive function.

Multimodal magnetic resonance imaging (MRI) techniques are widely employed to assess alterations in brain structural and function, crucial for the early diagnosis of CI in individuals with CKD (Steinbach and Harshman 2022). Structural MRI investigations have indicated a significant decrease in cortical gray matter volume (GMV) in CKD, closely associated with cognitive deficits (Yuan et al. 2023; Tsuruya et al. 2015). Complementing structural assessments, perfusion MRI provides insights into cerebral blood flow (CBF) dynamics, highlighting the reductions of CBF strongly correlated with the severity of cognitive decline in CKD (Lin et al. 2023, Li et al. 2024). Moreover, resting‐state functional MRI (rs‐fMRI) has uncovered disruptions in functional connectivity (FC) within critical brain networks, offering deeper insights into the neural mechanisms underlying CI in CKD (Y. F. Wang et al. 2019; Yang et al. 2024). However, most studies have focused on patients with end‐stage CKD, leaving a gap in understanding brain changes in those with early‐stage CKD. This highlights the urgent need to clarify brain structural changes, abnormal perfusion, and functional disruption at different stages of CKD, as well as their relationship with CI. Specifically, identifying the imaging features of abnormal brain regions associated with CI in patients with early‐stage CKD could help in early identification and intervention strategies for CI, potentially preventing its progression to irreversible dementia (Pepin et al. 2023). We hypothesized that GMV, CBF, and FC differ across CKD stages and that these indicators are closely linked to changes in cognitive function.

In this study, we aim to clarify abnormal brain regions exhibiting altered GMV and CBF across different stage of CKD, utilizing these co‐changed brain regions as seed points to illustrate abnormal seed‐based FC in whole brain and to investigate their correlation with neuropsychological test scores in patients with CKD.

## 2 Materials and Methods

### 2.1 Participants

From October 2020 to November 2023, a total of 142 patients with CKD from the Nephrology Department of Beijing Friendship Hospital, Capital Medical University, and 66 healthy controls (HCs) from local communities were consecutively enrolled in this study. The research adhered to the Declaration of Helsinki (as revised in 2013) and received approval from the Medical Ethics Committee of Beijing Friendship Hospital, Capital Medical University. Written informed consent was obtained from each participant prior to their enrollment in the study. The inclusion criteria for patients with CKD were the following: (1) diagnosed by professional nephrologist as glomerulonephritis; (2) age 18–65 years; the exclusion criteria of all participants included the following: (1) a history of dialysis treatment; (2) heart failure; (3) diabetic nephropathy, hypertensive kidney damage or unknown kidney disease; (4) neurological disorders, such as brain tumor, stroke, and brain trauma; (5) psychiatric disorders; (6) other systemic diseases, such as autoimmune diseases (e.g., lupus, rheumatoid arthritis), infectious diseases (e.g., HIV, tuberculosis), and systemic inflammatory disorders; (7) obvious head movement during MR scanning; and (8) MRI contraindications. The HCs did not have a history of kidney disease, and all participants were right‐handed.

Cognitive scale assessments and blood biochemical tests were conducted for all patients with CKD prior to the scanning. The Montreal Cognitive Assessment (MoCA) scale was utilized to evaluate cognitive abilities in patients with CKD. Demographic information was collected for all participants.

### 2.2 Magnetic Resonance Imaging Data Acquisition

MRI was performed on a 3T MR scanner (Discovery MR750 W, General Electric, Milwaukee, Wisconsin, USA) equipped with an eight‐channel phased array coil. During the scan, all participants were asked to lie in a supine position, with foam padding employed to minimize head movement. High‐resolution T1‐weighted images were obtained using the three‐dimensional brain volume imaging (3D BRAVO) sequence, achieving a structural image with an isotropic spatial resolution. The following parameters were used: repetition time (TR) = 8.8 ms, echo time (TE) = 3.5 ms, inversion time = 450 ms, field of view (FOV) = 240 × 240 mm^2^, matrix size = 256 × 256, axial slices = 196, slice thickness = 1 mm, flip angle (FA) = 15°, and acquisition time = 4 min 36 s. The 3D pseudo‐continuous arterial spin‐labeling (3D‐pCASL) sequence was performed for perfusion imaging with the following parameters: TR = 4844 ms, TE = 10.5 ms, FOV = 240 × 240 mm^2^, matrix size = 128 × 128, axial slices = 36, slice thickness = 4 mm, post‐labeling delay (PLD) times = 2025 ms, number of excitations = 3, spiral‐in readout = 8 arms×512 samples, and acquisition time = 5 min. The CBF maps were extracted from the original ASL data. The resting‐state functional data were obtained as the following parameters: TR = 2000 ms, TE = 35 ms, FOV = 240 × 240 mm^2^, matrix size = 64 × 64, axial slices = 28, slice thickness = 5 mm, time points = 200, FA = 90°, and acquisition time = 6 min 40 s.

Before each scan, participants were instructed to relax with their eyes closed, not think of anything, and avoid falling asleep. Soft foam padding and earplugs were provided to minimize head motion, while earplugs also helped reduce scanner noise. All patients with CKD were required to complete an MRI scan prior to the operation of arteriovenous fistula.

### 2.3 GMV Images Segmentation and Analysis

The data preprocessing methods for calculating GMV were described in our previous study (Yang et al. 2024). Voxel‐based morphometry (VBM) processing was performed using the MATLAB‐based (R2018b; MathWorks) Statistical Parametric Mapping software (SPM12) and the SPM12‐based standard pipeline of a computational anatomy toolbox (CAT12). First, the Digital Imaging and Communications in Medicine format of the MRI T1 raw image data acquired by the 3D‐BRAVO sequence was converted to the Neuroimaging Informatics Technology Initiative (NIfTI) format. Subsequently, all attempted images were assessed for quality layer by layer, and images with large artifacts were rejected. For the segmentation of GMV images, first, the T1‐weighted images were normalized to Montreal Neurological Institute (MNI) space using the Diffeomorphic Anatomical Registration Through the Exponentiated Lie (DARTEL) algebra to enable the analysis of group data. After normalizing the T1‐weighted images, tissue probability maps were used to segment gray matter, white matter, and cerebrospinal fluid tissues. Finally, the GMV images were smoothed to reduce spatial noise using a 6 mm×6 mm×6‐mm full‐width half maximum (FWHM) isotropic Gaussian kernel.

### 2.4 Cerebral Blood Flow Calculation and Analysis

The methods for calculating CBF were described in our previous studies (Wang et al. 2020, 2022, 2020, 2022). All ASL images underwent visual inspection to identify artifacts or major anatomical abnormalities, and quality controls were performed to exclude unqualified results. The ASL difference image was calculated by subtracting the label images from the control images using the single‐compartment model. CBF images were generated from the proton density‐weighted reference and ASL difference images. These CBF maps were then converted to NIfTI format, with each image being assessed for quality layer by layer. The CBF images with large artifacts were rejected. Using the DARTEL technique of the SPM12 software, the CBF maps in the individual spaces of all participants were individually normalized to the MNI standard space. After normalization, the quality of the CBF images of all participants was assessed again, and severely distorted CBF images were removed for the next step of analysis. All data were standardized using the mean division method of the Data Processing Assistant for Resting‐State fMRI (DPARSF) advanced edition software package (Yan et al. 2016) to increase parameter sensitivity. Finally, all standardized CBF images were spatially smoothed using an isotropic Gaussian kernel with a 6 mm × 6 mm×6 mm FWHM to improve the signal‐to‐noise ratio of the CBF images.

### 2.5 Functional MRI Data Preprocessing and Analysis

The data preprocessing methods for calculating FC were described in our previous study (Yang et al. 2024). The DPARSF Advanced Edition software package (Data Processing & Analysis of Brain Imaging, http://rfmri.org/dpabi) was utilized for preprocessing the functional imaging data. The first 10 volumes for each participant were excluded from the acquisition of 200 volumes, and temporal differences between slices were corrected in 190 volumes. Subsequently, head motion was corrected by realignment, and participants with head motion exceeding 3.0 mm were excluded. A nuisance regression analysis was performed with the motion parameters from the Friston‐24 model, the white matter, cerebral spinal fluid, and global signals as covariates. Coregistration step was then performed between the individual T1 and mean functional images. The acquired T1 images were then segmented and spatially normalized to the MNI space using the DARTEL algebra technique. The normalized functional images were filtered using a bandpass filter with a frequency range of 0.01–0.10 Hz. Significantly different regions based on changes in GMV and CBF were further selected as seed points for FC analysis. After the above analysis, we defined the left fusiform and right fusiform as seed points for subsequent FC analysis. FC maps were derived from the correlation between the mean time series of the seed points and the time series of the voxels within the total brain cortices. Fisher's Z transformation was used to ensure that the data were normally distributed. Spatial smoothing was performed on the FC maps with a 6 mm×6 mm×6‐mm FWHM isotropic Gaussian kernel.

### 2.6 Statistical Analysis

All statistical analyses were performed using SPSS 26.0 software (SPSS Inc., Chicago, IL, USA) and SPM12 package (https://www.fil.ion.ucl.ac.uk/spm/). A significance level of *p* < 0.05 was established for two‐sided tests. The Shapiro–Wilk test was employed to assess the normality of the clinical data. For quantitative variables, normally distributed data were expressed as means ± standard deviations, while non‐normally distributed variables were presented as medians with ranges. Categorical variables were evaluated using the *χ* ^2^ test or Fisher's exact test. Differences in age, sex, and education level among the HC, CKD 1–3a, and CKD 3b–5 groups were assessed using one‐way analysis of variance (ANOVA) for normally distributed data, and the Kruskal–Wallis test for non‐normally distributed data. Difference in blood biochemical indexes between the CKD 1–3a and CKD 3b–5 groups were analyzed with independent sample *t*‐tests for normally distributed data and Mann–Whitney *U* test for non‐normally distributed data.

The group differences in GMV, CBF, and FC values were performed using voxel‐wise one‐way ANOVA with age, sex, and TIV as covariates. A significant threshold of voxel‐wise *p* < 0.001 uncorrected was applied to all MRI data analysis, along with cluster‐level *p* < 0.05 false discovery rate (FDR) or Gaussian random field (GRF) correction. Pearson's or Spearman's correlation analysis was used to determine the correlations between clinical characteristics and imaging parameters (GMV, CBF, and FC), adjusting for age, sex, education level, and hemoglobin. Positive and negative coefficients (*r*) indicate positive and negative correlations. The Bonferroni method was employed for multiple comparisons.

## 3 Results

### 3.1 Patient Demographics and Clinical Characteristics

**FIGURE 1:**
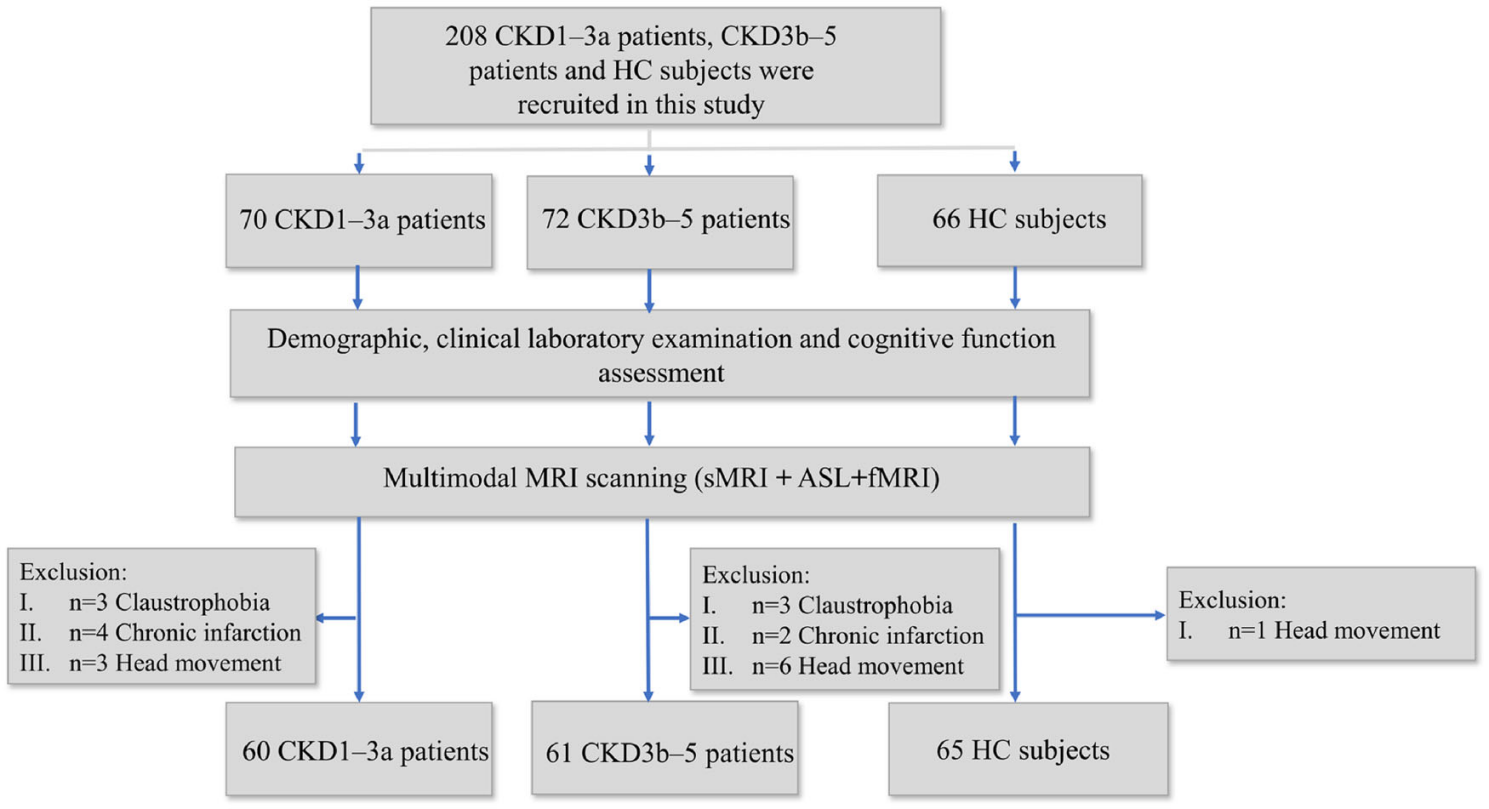
The patient enrollment flowchart. ASL, arterial spin labeling; CKD, chronic kidney disease; CKD 1–3a, patients with Stage 1–3a chronic kidney disease; CKD 3b–5, patients with Stage 3b–5 chronic kidney disease; fMRI, functional magnetic resonance imaging; HC, healthy control; sMRI, structural MRI.

Two hundred and eight participants (66 age‐, sex‐, and education degree matched HCs, 70 patients with CKD 1–3a, and 72 patients with CKD 3b–5) were consecutively recruited for this study. The patient enrollment flowchart is shown in Figure 1. Ten patients with CKD 1–3a were excluded due to claustrophobia (*n* = 3), chronic infarction (*n* = 4), and headache movement (*n* = 3). Eleven patients with CKD 3b–5 were excluded due to claustrophobia (*n* = 3), chronic infarction (*n* = 2), and headache movement (*n* = 6). One HC was excluded due to headache movement. A total of 186 participants, comprising 65 HCs, 60 patients with CKD 1–3a, and 61 patients with CKD 3b–5, were included in this study. The number of patients in each stage of CKD was as follows: CKD 1 (*n* = 25), CKD 2 (*n* = 26), CKD 3a (*n* = 11), CKD 3b (*n* = 5), CKD 4 (*n* = 1), and CKD 5 (*n* = 55). The demographic and clinical characteristics of the three groups are summarized in Table 1. No significant differences were observed among the groups regarding age, sex, or education degree (all *p* > 0.05). Patients with CKD 3b–5 exhibited significantly higher levels of urea, creatinine, blood albumin, and hemoglobin compared to those with CKD 1–3a (all *p* < 0.01). They also had lower eGFR values and elevated phosphorus levels (all *p* < 0.001). Furthermore, patients with CKD 3b–5 scored lower on the Montreal Cognitive Assessment (MoCA) compared to those with CKD 1–3a (*p* = 0.001).

**TABLE 1:** Demographic and clinical characteristics of the subjects.

	CKD 1–3a (*n* = 60)	CKD 3b–5 (*n* = 61)	HC (*n* = 65)	*p*‐value
Age (years)^a^	48.5 (37.0, 60.0)	52.0 (43.5, 60.0)	52.0 (43.0, 56.0)	0.511^b^
Sex (male/female)	41/19	41/20	37/28	0.337^c^
Education (years)^a^	12.0 (9.8, 16.0)	12.0 (12.0, 15.0)	12.0 (12.0, 15.0)	0.413^b^
Urea (mmol/L)^a^	6.0 (4.6, 7.3)	26.6 (20.1, 33.9)	NA	**< 0.001** ^d^
Creatinine (µmol/L)^e^	89.6 ± 26.3	673.3 ± 295.6	NA	**< 0.001** ^f^
eGFR (ml/min/1.73m^2^)	84.4 (65.7, 100.6)	6.6 (4.9, 9.8)	NA	**< 0.001** ^d^
albumin (g/L)^a^	32.4 (27.3, 37.9)	36.3 (32.0, 39.2)	NA	**0.015** ^d^
Hypertension history (%)	19 (31.7%)	11 (18.0%)	NA	**0.095** ^g^
Uric Acid (µmol/L)^a^	401.3 (338.9, 474.6)	396.1 (342.2, 516.1)	NA	0.789^d^
Phosphorus (mmol/L)^a^	1.2 (1.1, 1.4)	2.0 (1.5, 2.4)	NA	**< 0.001** ^d^
Calcium (mmol/L)^e^	2.2 ± 0.2	2.1 ± 0.2	NA	0.107^f^
Hemoglobin (g/L)^e^	132.1 ± 19.1	101.3 ± 20.5	NA	**< 0.001** ^f^
MoCA^e^	26.0 (24.0, 28.0)	24.0 (20.0, 26.0)	NA	**0.001** ^d^

Abbreviations: CKD 1–3a, patients with Stage 1–3a chronic kidney disease; CKD 3b–5, patients with Stage 3b–5 chronic kidney disease; eGFR, glomerular filtration rate; HC = healthy control; MoCA = Montreal Cognitive Assessment; NA = not applicable.

^a^ Data are presented as median (25th–75th percentile).

^b^ Kruskal–Wallis test

^c^ *χ* ^2^ test.

^d^ Mann–Whitney *U*‐test

^e^ Data are presented as mean ± SD.

^f^ Two‐sample *t*‐tests.

^g^ Fisher's exact test.

^h^ P<0.05.

### 3.2 GMV Differences Among the Three Groups

**FIGURE 2:**
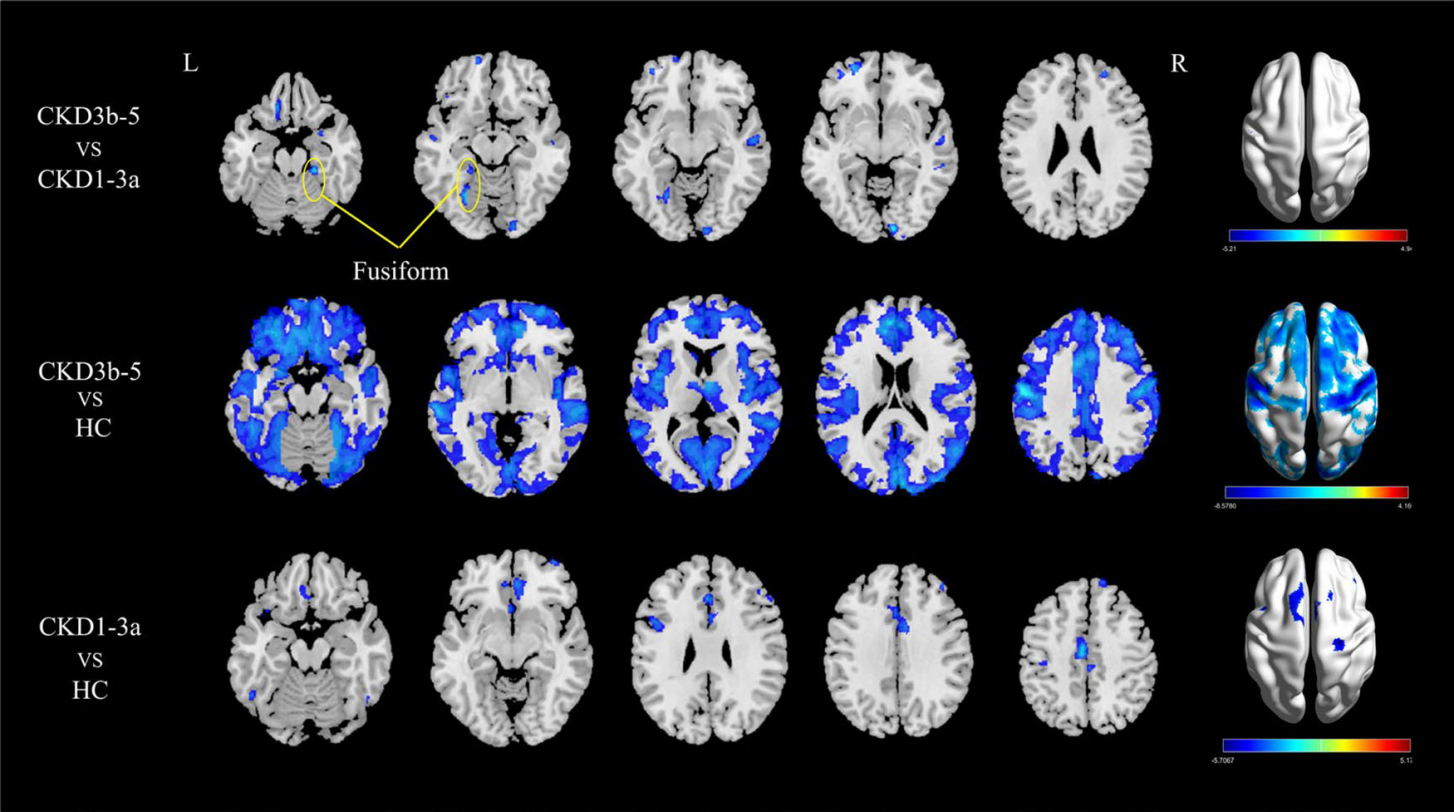
Voxel‐based GMV analysis among CKD 1–3a patients, CKD 3b–5 patients, and HC (*p* < 0.05, FDR corrected). The results were shown on the study‐wise magnitude template in the MNI coordinate system. CKD, chronic kidney disease; CKD 1–3a, patients with Stage 1–3a chronic kidney disease; CKD 3b–5, patients with Stage 3b–5 chronic kidney disease; FDR, false discovery rate; GMV, gray matter volume; HC, healthy control; MNI, Montreal Neurological Institute;.

As shown in Table 2, the CKD 3b–5 group exhibited significantly reduced GMV in the bilateral fusiform gyrus, right middle frontal gyrus (MFG.R), and bilateral middle temporal gyrus (MTG.L and MTG.R) compared to the CKD 1–3a group (*p* < 0.05, cluster‐level FDR‐corrected). Moreover, the CKD 3b–5 group manifested decreased GMV in the bilateral fusiform gyrus, thalamus, and hippocampus when compared to the HC group (*p* < 0.05, cluster‐level FDR‐corrected). Furthermore, the CKD 1–3a group displayed reduced GMV in the left inferior temporal gyrus (ITG.L), right middle frontal gyrus, bilateral superior frontal gyrus, dorsolateral (SFGdor.L and SFGdor.R), as well as the bilateral median cingulate and paracingulate gyri (DCG.L and DCG.R) compared to the HC group (*p* < 0.05, cluster‐level FDR‐corrected) (Figure 2).

**TABLE 2:** Brain regions exhibiting GMV differences among the three groups.

Brain regions	Cluster size	MNI coordinate			Peak *t*‐value
		*x*	*y*	*z*	
**CKD 3b–5 < CKD 1–3a**					
Left fusiform	183	−24	−62	−13	−5.46
Right fusiform	101	24	−30	−21	−5.53
Right middle frontal gyrus	89	25	49	25	−4.78
Left middle temporal gyrus	74	−54	−61	13	−4.90
Right middle temporal gyrus	68	54	−31	0	−4.45
**CKD 3b–5< HC**					
Left fusiform	2417	−28	−49	−14	−5.03
Right fusiform	2901	27	−48	−14	−5.73
Left thalamus	417	−9	−9	11	−3.84
Right thalamus	1418	7	−14	11	−5.47
Left hippocampus	435	−34	−27	−9	−3.55
Right hippocampus	212	38	−26	−9	−3.24
**CKD 1–3a < HC**					
Left inferior temporal gyrus	46	−51	−55	−16	−4.77
Right middle frontal gyrus	69	40	40	36	−4.60
Left superior frontal gyrus, dorsolateral	379	−18	15	63	−4.64
Right superior frontal gyrus, dorsolateral	61	18	21	57	−4.67
Left median cingulate and paracingulate gyri	670	−4	17	35	−4.07
Right median cingulate and paracingulate gyri	355	4	4	35	−4.61

*Note*: Brain regions exhibiting GMV differences among groups. CKD 1–3a, patients with Stage 1–3a chronic kidney disease; CKD 3b–5, patients with Stage 3b–5 chronic kidney disease; GMV, gray matter volume; HC, healthy control; MNI, Montreal Neurological Institute.

### 3.3 CBF Differences Among the Three Groups

**FIGURE 3:**
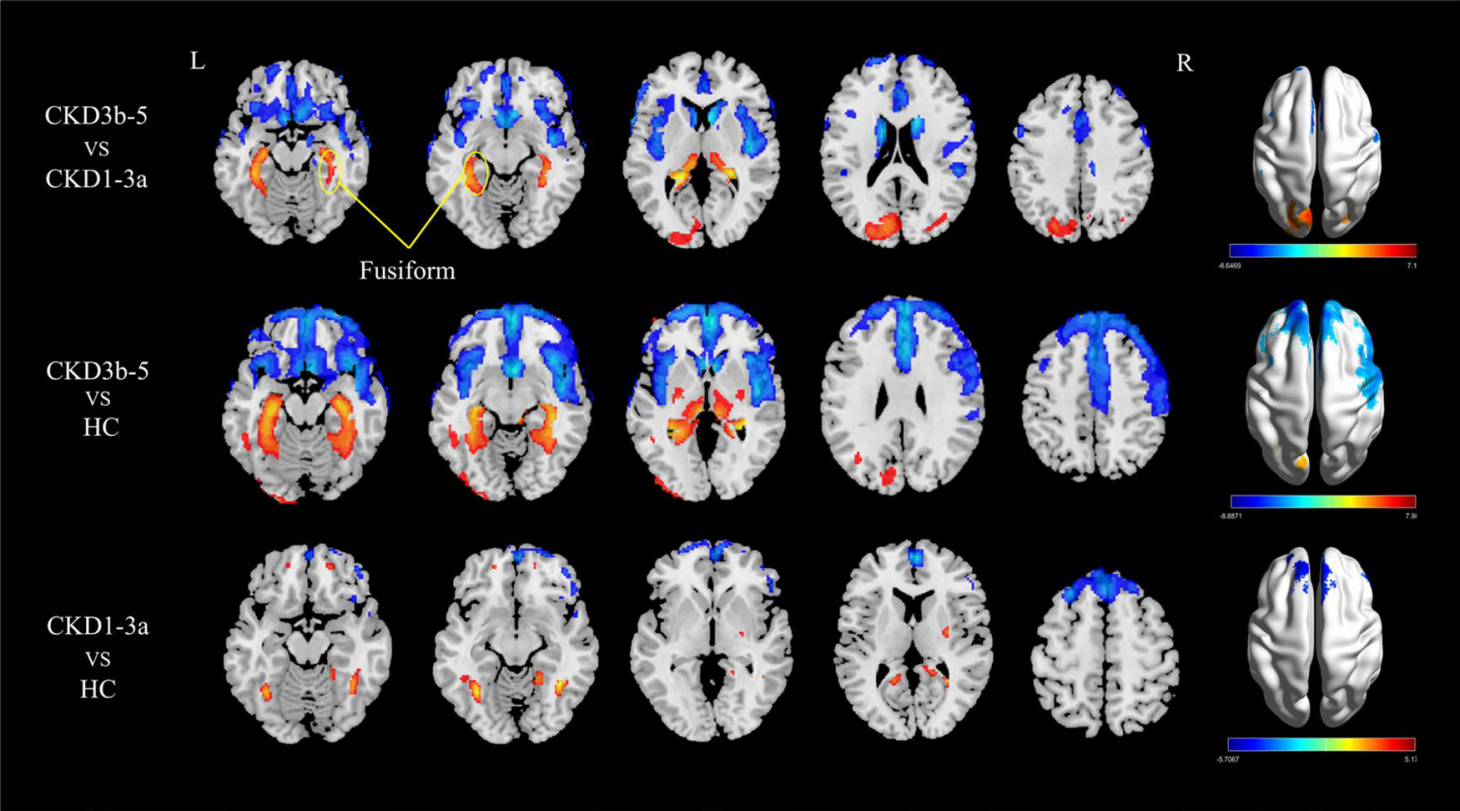
Voxel‐based CBF analysis among CKD 1–3a patients, CKD 3b–5 patients, and HC (*p* < 0.05, FDR corrected). The results were shown on the study‐wise magnitude template in the MNI coordinate system. CBF, cerebral blood flow; CKD, chronic kidney disease; CKD 1–3a, patients with Stage 1–3a chronic kidney disease; CKD 3b–5, patients with Stage 3b–5 chronic kidney disease; FDR, false discovery rate; HC, healthy control; MNI, Montreal Neurological Institute;.

As presented in Table 3, the CKD 3b–5 group exhibited higher CBF in the bilateral fusiform gyrus, thalamus, and hippocampus compared to the CKD 1–3a and HC groups, while showing lower CBF in the bilateral caudate nucleus, and insula (*p* < 0.05, cluster‐level FDR‐corrected). In addition, the CKD 1–3a group showed increased CBF in the bilateral fusiform gyrus, and left precuneus, with lower CBF in the MFG.L, SFGdor.L, and SFGdor.R compared with the HC group (*p* < 0.05, cluster‐level FDR‐corrected). The voxel‐based CBF analysis among the three groups is illustrated in Figure 3.

**TABLE 3:** Brain regions exhibiting CBF differences among the groups.

Brain regions	Cluster size	MNI coordinate			Peak *t*‐value
		*x*	*y*	*z*	
**CKD 3b–5 > CKD 1–3a**					
Left fusiform	318	−20	−40	8	7.16
Right fusiform	57	34	−43	−13	4.20
Left thalamus	223	−8	−24	6	4.98
Right thalamus	220	12	−24	6	4.26
Left hippocampus	385	−28	−18	−14	5.15
Right hippocampus	313	28	−38	4	6.77
Left middle occipital gyrus	540	−18	−86	20	3.64
Right middle occipital gyrus	173	38	−72	30	3.77
**CKD 3b–5 < CKD 1–3a**					
Left median cingulate and paracingulate gyri	312	−2	19	36	−4.47
Right median cingulate and paracingulate gyri	269	2	5	38	−3.45
Left caudate nucleus	604	−10	11	12	−4.19
Right caudate nucleus	568	9	13	12	−5.90
Left insula	1083	−38	12	0	−4.60
Right insula	1259	42	−4	−3	−5.67
**CKD 3b–5 > HC**					
Left hippocampus	597	−30	−20	−14	7.00
Right hippocampus	490	30	−36	4	7.98
Left fusiform	615	−32	−47	−14	5.04
Right fusiform	733	35	−38	−14	5.07
Left thalamus	495	−15	−22	1	3.96
Right thalamus	588	13	−19	1	3.73
**CKD 3b–5< HC**					
Left caudate nucleus	465	−8	17	−6	−4.16
Right caudate nucleus	511	5	14	−6	−7.47
Left insula	1216	−40	18	−7	−5.27
Right insula	1175	42	6	−7	−5.86
Left middle frontal gyrus	833	−31	59	11	−3.90
Right middle frontal gyrus	2005	33	59	10	−3.10
**CKD 1–3a> HC**					
Left precuneus	118	−16	−50	18	4.94
Left fusiform gyrus	184	−34	−58	−10	5.03
Right fusiform gyrus	164	20	−42	−14	3.96
**CKD 1–3a < HC**					
Left middle frontal gyrus	249	−28	29	58	−4.38
Left superior frontal gyrus, dorsolateral	478	−13	47	45	−4.26
Right superior frontal gyrus, dorsolateral	138	21	29	58	−3.79

*Note*: Brain regions exhibiting CBF differences among groups. CBF, cerebral blood flow; CKD 1–3a, patients with Stage 1–3a chronic kidney disease; CKD 3b–5, patients with Stage 3b–5 chronic kidney disease; HC, healthy control; MNI, Montreal Neurological Institute;.

### 3.4 Seed‐Based FC Differences Among the Three Groups

**FIGURE 4:**
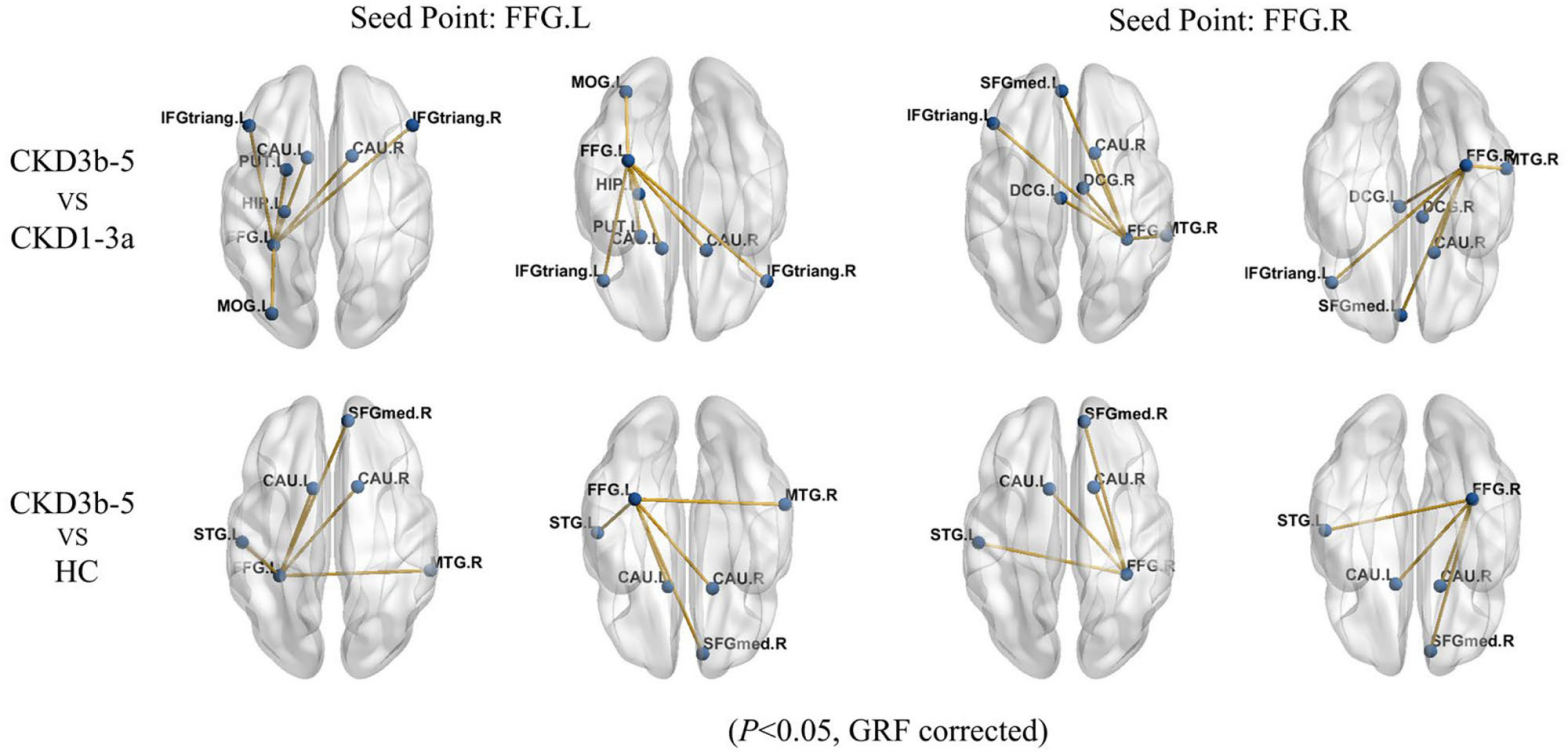
Voxel‐based FC analysis among CKD 1–3a patients, CKD 3b–5 patients and HC (*p* < 0.05, GRF corrected). The results were shown on the study‐wise magnitude template in the MNI coordinate system. CAU.L, left caudate nucleus; CAU.R, right caudate nucleus; CKD, chronic kidney disease; CKD 1–3a, patients with Stage 1–3a chronic kidney disease; CKD 3b–5, patients with Stage 3b–5 chronic kidney disease; FC, functional connectivity; FFG.L, left fusiform gyrus; FFG.R, right fusiform gyrus; GRF, Gaussian random field; HC, healthy control; HIP. L, left hippocampus; IFGtriang.L, left inferior frontal gyrus, triangular part; IFGtriang.R, right inferior frontal gyrus, triangular part; MNI, Montreal Neurological Institute; MOG.L, left middle occipital gyrus; MTG.R, right middle temporal gyrus; PUT.L, left putamen; SFGmed.R, right superior frontal gyrus, medial; STG.L, left superior temporal gyrus.

On the basis of the above results, abnormal GMV and CBF in the bilateral fusiform gyrus were observed among the three groups. We defined the bilateral fusiform gyrus as a co‐changed brain region. And the bilateral fusiform gyrus were chosen as seed regions for FC analyses in the present study. The results for the seed point located in the bilateral fusiform gyrus (FFG.L‐FC, and FFG.R‐FC) are presented in Table 4 and Figure 4. The CKD 3b–5 group exhibited significantly lower FFG.L‐FC in the bilateral inferior frontal gyrus, triangular part (IFGtriang.L and IFGtriang.R), MOG.L, CAU.L, CAU.R, left putamen, and left hippocampus compared to the CKD 1–3a group (*p* < 0.05, cluster‐level FDR‐corrected). And the CKD 3b–5 group exhibited significantly lower FFG.R‐FC in the IFGtriang.L, DCG.L, DCG.R, SFGmed.L, CAU.R, and MTG.R compared to the CKD 1–3a group (*p* < 0.05, cluster‐level FDR‐corrected).

**TABLE 4:** Brain regions exhibiting FC differences among groups.

Brain regions	Cluster size	MNI coordinate			Peak *t*‐value
		x	y	z	
**Seed point: left fusiform gyrus**					
** CKD 3b–5 < CKD 1–3a**					
Right inferior frontal gyrus, triangular part	33	39	33	18	−4.09
** L**eft inferior frontal gyrus, triangular part	67	−38	21	25	−4.42
Left middle occipital gyrus	44	−30	−75	15	−4.59
Left caudate nucleus	24	−8	18	−6	−4.01
Right caudate nucleus	38	12	18	−3	−4.84
Left putamen	24	−22	19	−2	−3.87
Left hippocampus	62	−22	−28	−6	−3.71
** CKD 3b–5< HC**					
Right middle temporal gyrus	44	45	−3	−27	−3.98
Left caudate nucleus	28	−15	24	−6	−4.41
Right caudate nucleus	25	15	21	−6	−4.25
Right superior frontal gyrus, medial	28	12	47	1	−4.64
Left superior temporal gyrus	62	−42	−24	6	−4.88
**Seed point: right fusiform gyrus**					
** CKD 3b–5 < CKD 1–3a**					
Left median cingulate and paracingulate gyri	32	9	11	40	−4.44
Right median cingulate and paracingulate gyri	39	−6	12	27	−3.81
Left superior frontal gyrus, medial	95	−9	54	9	−4.25
Right caudate nucleus	36	24	24	0	−4.75
Left inferior frontal gyrus, triangular part	55	−39	18	21	−4.60
Right middle temporal gyrus	175	66	−27	−3	−4.94
** CKD 3b–5 < HC**					
Right superior frontal gyrus, medial	71	12	58	15	−3.78
Left superior temporal gyrus	96	−42	−24	6	−5.49
Left caudate nucleus	28	−9	−18	−9	−4.08
Right caudate nucleus	39	15	24	−6	−4.75

*Note*: Brain regions exhibiting FC differences among groups (*p* < 0.05, GRF corrected). CKD 1–3a, patients with Stage 1–3a chronic kidney disease; CKD 3b–5, patients with Stage 3b–5 chronic kidney disease; FC, functional connectivity; GRF, Gaussian random field; HC, healthy control; MNI, Montreal Neurological Institute;.

The CKD 3b–5 group had lower FFG.L‐FC and FFG.R‐FC in the SFGmed.R, left superior temporal gyrus (STG.L), CAU.L, and CAU.R compared to the HC group (*p* < 0.05, cluster‐level FDR‐corrected). No significant differences in FFG.L‐FC and FFG.R‐FC were found between the HC and CKD 1–3a groups.

### 3.5 Correlations Analysis Between the MoCA Scores and Imaging Parameters

**FIGURE 5:**
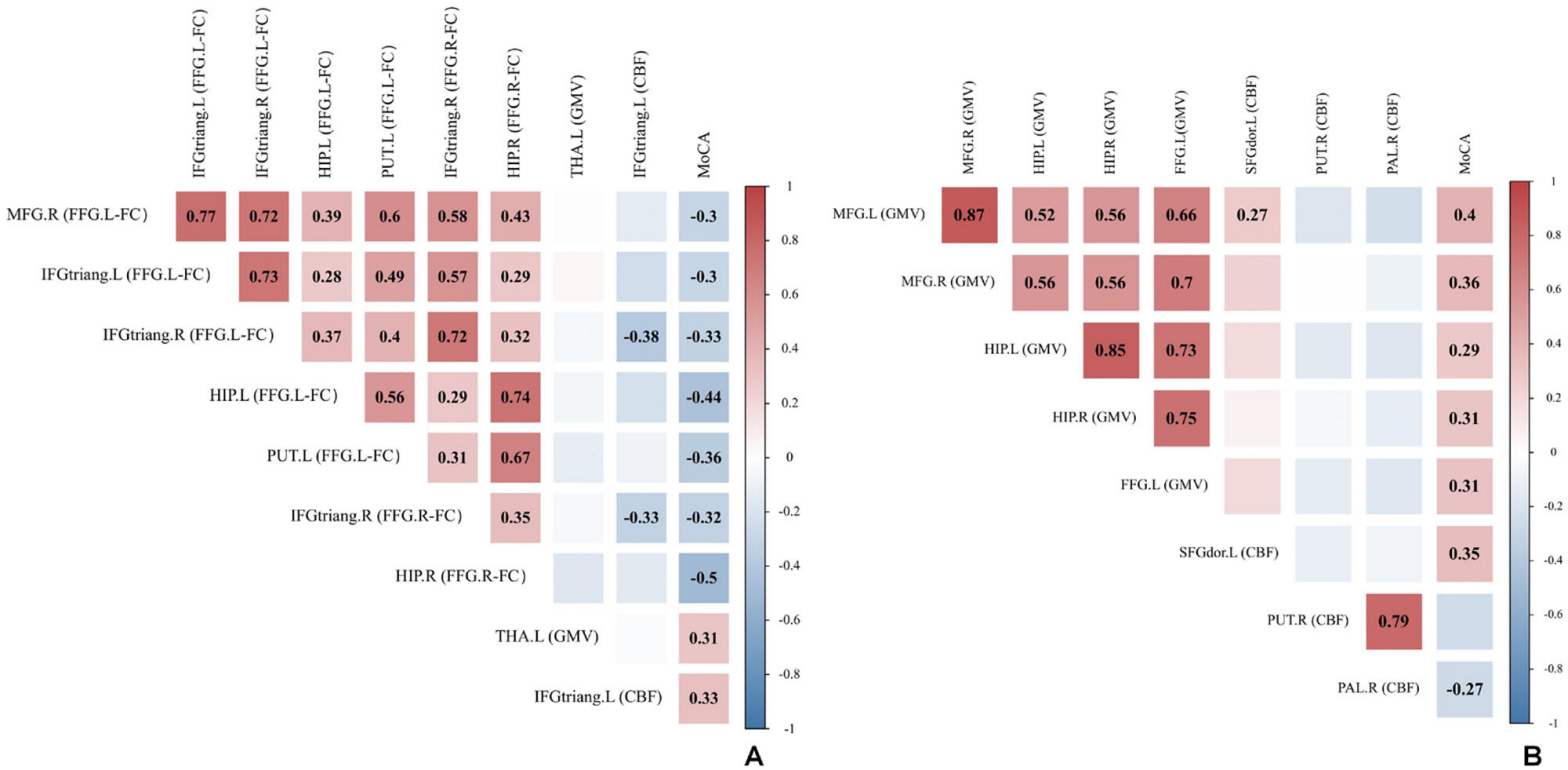
Correlation between MoCA scores and FC, GMV, and CBF in patients with CKD 1–3a (A). Correlation between MoCA scores and FC, GMV, and CBF in patients with CKD 3b–5 (B). The color scale from blue to red represents the *r*‐value from −1 to 1. The values of the correlation coefficients marked in the graph are all statistically significant (*p* < 0.05). CKD, chronic kidney disease; CKD 1–3a, patients with Stage 1–3a chronic kidney disease; CKD 3b–5, patients with Stage 3b–5 chronic kidney disease; FC, functional connectivity; FFG, fusiform gyrus; HIP, hippocampus; IFGtriang, inferior frontal gyrus, triangular part; L, left; MRF, middle frontal gyrus; PAL, lenticular nucleus, pallidum; PUT, lenticular nucleus, putamen; R, right; SFGdor, superior frontal gyrus, dorsolateral; THA, thalamus.

In the CKD 1–3a group, a negative correlation was observed between MoCA scores and FFG.L–FC in the MFG.R (*r* = −0.301, *p* = 0.024), IFGtriang.L (*r* = −0.299, *p* = 0.025), IFGtriang.R (*r* = −0.328, *p* = 0.014), HIP.L (*r* = −0.445, *p* = 0.001) and PUT.L (*r* = −0.362, *p* = 0.006), Also, a negative correlation was found between MoCA scores and FFG.R–FC in the IFGtriang.R (*r* = −0.323, *p* = 0.015) and HIP.R (*r* = −0.503, *p* < 0.001). Conversely, the GMV in the THA.L (*r* = 0.307, *p* = 0.022) and CBF in the IFGtriang.L (*r* = 0.331, *p* = 0.013) were positively correlated with the MoCA scores after adjustments for age, sex, education degree, and hemoglobin. The results of correlation analysis for CKD 1–3a group are shown in Figure 5A.

In the CKD 3b–5 group, no significant correlations were found between the MoCA scores and FC based on FFG.L and FFG.R in any brain regions (all *p* > 0.05). The GMV in the MFG.L (*r* = 0.401, *p* = 0.002), MFG.R (*r* = 0.363, *p* = 0.005), HIP.L (*r* = 0.288, *p* = 0.030), HIP.R (*r* = 0.307, *p* = 0.020), and FFG.L (*r* = 0.312, *p* = 0.018), as well as the CBF in the SFGdor.L (*r* = 0.346, *p* = 0.008) were positively correlated with the MoCA scores. Conversely, the CBF in the right lenticular nucleus, pallidum (PAL.R) (*r* = −0.270, *p* = 0.042) showed negative correlations with MoCA scores after adjustments for age, sex, education degree, and hemoglobin. The results of correlation analysis for the CKD 3b–5 group are shown in Figure 5B.

## 4 Discussion

In the present study, we observed that all patients with CKD exhibited decreased GMV compared to HCs in some brain regions. Also, patients with CKD 3b–5 displayed a significant reduction in GMV compared to those with CKD 1–3a, specifically in the bilateral fusiform gyrus. Meanwhile, patients with CKD 3b–5 demonstrated increased CBF in the bilateral fusiform gyrus compared to those with CKD 1–3a. The patients with CKD 3b–5 showed decreased FC between the bilateral fusiform gyrus and some regions related to cognitive function, including IFGtriang.L, IFGtriang.R, MOG.L, CAU.L, CAU.R, left putamen, left hippocampus, DCG.L, DCG.R, SFGmed.L, and MTG.R, compared to those with CKD 1–3a. These findings could enhance our comprehension of the neural mechanisms that contribute to cognitive decline in CKD.

Cerebral atrophy is a common phenomenon in the progression of CKD, which may underlie the cognitive decline observed in these patients (Tsuruya and Yoshida 2024). Our study found significant reductions in GMV in patients with CKD compared to HCs, particularly in the bilateral fusiform gyrus. Our findings are partially aligned with previous studies that reported reduced GMV in patients with CKD (Yang et al. 2024, Maki et al. 2023; Cercignani et al. 2015). The reduced GMV could be attributed to several factors, including uremia‐related toxins, metabolic disturbances, and the cumulative effects of inflammation and oxidative stress (Drew et al. 2019), which may collectively impair neuronal integrity and function. Fusiform gyrus is known for its critical role in cognitive functions such as visual object recognition and language processing (Weiner and Zilles 2016; Chen et al. 2022). The cerebral atrophy in fusiform gyrus may contribute to observed CIs through various mechanisms such as changes in neural connectivity or synaptic plasticity. Strikingly, the GMV reduction was more pronounced in patients with CKD 3b–5 compared to those in earlier Stages 1–3a, suggesting that the severity of the disease was related to the degree of brain atrophy. This study highlights the importance of early detection and intervention for preventing brain atrophy and CI in patients with CKD. Previous studies have highlighted the significant impact of recombinant human erythropoietin (rHuEPO), empagliflozin, vitamin D, and sodium‐glucose cotransporter‐2 inhibitors (SGLT2i) in mitigating CI (Barbieri et al. 2024, Mone et al. 2024; Cheng et al. 2016; Noel, Hougen, and Sood 2022). Future longitudinal studies are essential to investigate the effects of these therapies on brain structure as well as to assess their potential in improving cognitive outcomes in patients with different stages CKD.

Cerebral perfusion abnormalities are significant contributors to cognitive dysfunction in CKD (Berkhout‐Byrne et al. 2017). In our study, we observed that CKD 3b–5 group exhibited reduced CBF in the bilateral caudate nucleus and insula compared to HC and CKD 1–3a groups. This finding aligns with previous research (Lin et al. 2023). The caudate nucleus and insula are regions critically involved in cognitive processes such as attention, executive function, and emotional regulation. Hypoperfusion in these areas could lead to neuronal damage, central cholinergic dysfunction, and oxidative stress, all of which contribute to cognitive deficits (Daulatzai 2017). Thus, we speculate that decreased CBF in the caudate nucleus and insula may result in insufficient perfusion, potentially serving as a significant factor contributing to CI in CKD patients. In addition, the hemoglobin levels of patients with CKD 3b–5 were significantly reduced in this study, which may be closely associated with the decrease in CBF and the alteration in cognitive function by reducing oxygen delivery to the brain (Hoiland et al. 2016; Yu et al. 2024). The effects of hemoglobin levels on cerebral perfusion and cognitive function across different CKD stages warrants investigation in future studies with larger sample sizes. Interestingly, our study also found increased CBF in the bilateral fusiform gyrus, thalamus, and hippocampus in CKD 3b–5 group when compared to both HC and CKD 1–3a groups. This observation partially aligns with previous studies. For instance, a perfusion study by Cheng et al. using ASL to investigate CBF changes in patients with end‐stage renal disease (ESRD) who were undergoing long‐term peritoneal dialysis. They discovered that patients with ESRD exhibited a mean increase in CBF compared to HCs, and the increased CBF observed in the left hippocampus was associated with poorer executive function (Cheng et al. 2018). Our earlier research on non‐dialysis patients with Stage 5 CKD also revealed higher normalized CBF in the hippocampus and thalamus (H. Wang et al. 2023). While these studies identified similar CBF alterations within the hippocampus as ours did, our study was conducted on patients with Stages 1–5 CKD. Therefore, a large sample size study is still needed for verification in future research. In the current study, we made an interesting observation that the CKD 1–3a group exhibited significantly higher CBF levels within the fusiform gyrus compared to the HC group—a finding not previously reported. Earlier research on perfusion mainly concentrated on patients with ESRD and those undergoing hemodialysis. This observed hyperperfusion in early‐stages CKD could potentially indicate a compensatory response to early neuronal degeneration or could indicate maladaptive changes occurring (Gach et al. 2023). Such changes might serve as early biomarkers for cognitive decline in patients with CKD. Monitoring cerebral perfusion changes in regions such as the fusiform gyrus, thalamus, and hippocampus may provide valuable insights into early detection and management of CI in individuals with CKD.

In this study, FC with FFG as seed point are differed significantly among the three groups. The CKD 3b–5 group exhibited reduced FFG‐FC with prefrontal, temporal, and limbic lobes (including IFGtriang.L, IFGtriang.R, SFGmed.L, DCG.L, DCG.R, MTG.R, and HIP.L) compared to CKD 1–3a group. And the CKD 3b–5 group exhibited decreased FFG‐FC with prefrontal and temporal lobes (SFGmed.R, MTG.R, and STG.L) compared to HC group. These findings are partially aligned with previous studies (Ni et al. 2014; Qiu et al. 2014). Employing independent component analysis, Ni et al. discovered that patients with ESRD demonstrated reduced FC in the default mode network (DMN) in comparison to HCs. Our study results share some similarities; however, the differences may be attributed to variations in technology and grouping (Ni et al. 2014). The reduction in FC observed in advanced stages of CKD is likely a result of combined effects from decreased GMV and CBF which potentially impair functional integration within critical networks (Qiu et al. 2014). Notably, the affected areas are predominantly within the DMN, a key network for maintaining cognitive functions such as memory, attention, and self‐referential thinking (Smallwood et al. 2021). The disruption of FC within the DMN in patients with CKD 3b–5 indicated a degradation in network integrity, which may underlie the cognitive decline observed in these individuals. In addition, our study found no differences in FFG‐FC with cognitive‐related regions between early‐stage CKD and HCs. This finding suggested that brain functional networks, including DMN, remain relatively intact in patients with early‐stages CKD. This implied that brain structure and perfusion changes in CKD‐related CI may precede alterations in functional activity, highlighting GMV and CBF as potential imaging biomarkers for early detection of cognitive dysfunction in patients with CKD. As the disease progresses, brain structural and hemodynamic alterations may contribute to abnormal brain activity in CKD patients. Our findings offer new perspectives on the pathological mechanisms of CI and underscore the importance of early intervention in CKD.

In the present study, we observed that GMV in the MFG.L, MFG.R, HIP.L, and HIP.R, as well as CBF in the SFGdor.L, showed positive correlations with MoCA scores in the CKD 3b–5 group. This finding aligns with previous studies, suggesting that structural atrophy and reduced cerebral perfusion in these areas contribute significantly to CI in advanced‐stages CKD (Li et al. 2022; Bracko et al. 2021). Interestingly, we identified a negative correlation in the CKD 3b–5 group between CBF and MoCA scores in the PUT.R and PAL.R, both of which are components of the basal ganglia. The basal ganglia are essential for motor and cognitive functions, and elevated CBF in this region might indicate an effort to compensate for neuronal damage. Notably, there was no significant correlation found between MoCA scores and FFG‐FC in any brain region within the CKD 3b–5 group. In contrast, the CKD 1–3a group showed a negative correlation between MoCA scores and FFG‐FC in several regions, which was similar with previous research (Su et al. 2019). Increased FC may indicate a compensatory reaction in spontaneous brain activity in early‐stages CKD patients. However, this compensatory mechanism may become maladaptive and worsen CI as CKD progresses. In our previous study of non‐dialysis Stage 5 CKD patients, functional network parameters were found to be positively correlated with MoCA scores (Song et al. 2023). Another previous study reported microstructural and functional brain changes in ESRD undergoing dialysis, highlighting significant positive correlations between these changes and MoCA scores (Zheng et al. 2023). Future longitudinal studies are required to investigate the relationship between FC and cognitive function in advanced‐stages CKD. Moreover, several clinical factors, including hypertension, body mass index (BMI), eGFR, and albuminuria, have been shown to affect cognitive function in CKD patients (Berger et al. 2016; Santulli et al. 2024; Ookawara et al. 2022, Wilson et al. 2021). In this study, we observed a negative correlation between eGFR levels and regional CBF in the earlier stages of CKD (detailed in the supporting information), suggesting that the early‐stage CKD is associated with relatively preserved brain perfusion. However, as CKD progresses and eGFR levels decline, brain atrophy becomes more pronounced, potentially elevating the risk of CI in these patients.

This study had several limitations. First, it was a cross‐sectional study, incorporating only baseline MRI data from patients with CKD. Longitudinal follow‐up with clinical and imaging data is essential for a more comprehensive understanding of the changes in brain structure, perfusion, and functional activity within the same cohort over time. Second, lower eGFR values were observed in patients with Stage 3b–5. This is mainly since most patients were in Stage 5, with fewer in Stages 3b and 4 due to the rapid progression of the disease. As a result, there may be some bias in assessing the correlation between eGFR and imaging features (GMV and CBF) in these patients. Future studies should aim to increase the sample size of patients in Stages 3b and 4 to better explore the relationship between clinical indicators and imaging characteristics. Third, the hemoglobin levels may affect cerebral perfusion, potentially leading to bias in the results. To address this, we included hemoglobin levels as a covariate when analyzing the correlation between clinical cognitive scores and brain structural and functional imaging. In addition, this study did not evaluate the influence of clinical factors, such as BMI, diet habits, and living environment on CKD‐related CI. Previous research has highlighted the potential influence of BMI (Ookawara et al. 2022), high sodium intake (Borrelli et al. 2020), and environmental factors (Obrador et al. 2017) on patients with CKD, warranting further investigation. Furthermore, we employed the 116 brain regions’ AAL atlas in this study without examining subregions. Analyzing the cerebral structural and functional characteristics at different subregions is crucial, as they may possess distinct physiological functions that could influence the assessments of GMV, CBF, and FC. Finally, combining diffusion tensor imaging (DTI) and quantitative susceptibility mapping (QSM) with structural imaging, ASL, and fMRI could yield deeper insights into the mechanisms driving cognitive decline in CKD patients.

## 5 Conclusion

Brain structural and perfusion alterations may underlie the reduced FC between fusiform gyrus and cognitive‐related regions, providing potential neuroimaging evidence for the neuropathological mechanisms of CI in patients with CKD of different stages.

## Author Contributions

**Xiaoyan Bai**: writing–original draft, methodology, visualization, software, data curation. **Lijun Song**: methodology, visualization, writing–original draft, software, data curation. **Xu Liu**: data curation. **Wenbo Yang**: data curation. **Mingan Li**: data curation. **Boyan Xu**: data curation, software. **Zhenghan Yang**: methodology, conceptualization, supervision. **Zhen‐chang Wang**: supervision, methodology, conceptualization. **Hao Wang**: writing–review and editing, data curation, supervision, methodology, conceptualization.

## Study Approval Statement

The study was reviewed and approved by the Medical Ethics Committee of Beijing Friendship Hospital, Capital Medical University, approval number BFHHZS20220112.

## Consent

The subjects’ written informed consent was obtained to participate in the study.

## Conflicts of Interest

The authors declare no conflict of interest.

### Peer Review

The peer review history for this article is available at https://publons.com/publon/10.1002/brb3.70330

## Supporting information




**Figure S1**. Correlation between the eGFR level and imaging parameters (CBF and GMV) in patients with different CKD stages. eGFR, estimated glomerular filtration rate; IFGtriang.L, left inferior frontal gyrus, triangular part; MFG.L, left middle frontal gyrus.

## Data Availability

The data that support the findings of this study are not publicly available due to their containing information that could compromise the privacy of research participants but are available from the corresponding author HW upon reasonable request.
