# Supplementary material for: Cognitive Impairment in Chronic Kidney Disease Across Different Stages: The Role of Structural and Perfusion‐Driven Functional Connectivity Changes
Source: Brain Behav. 2025 Feb 17;15(2):e70330. doi: 10.1002/brb3.70330 (PMC11830999; doi:10.1002/brb3.70330)
Supplement: Supplementary file 1 — Figure S1. Correlation between the eGFR level and imaging parameters (CBF and GMV) in patients with different CKD stages. eGFR, estimated glomerular filtration rate; IFGtriang.L, left inferior frontal gyrus, triangular part; MFG.L, left middle frontal gyrus. [file BRB3-15-e70330-s001.docx]

**Correlations analysis between the eGFR level and imaging parameters**

In the CKD 1-3a group, a negative correlation was observed between the eGFR level and CBF in the IFGtriang.L (r=-0.266, P=0.048). In the CKD 3b-5 group, a positive correlation was found between the eGFR level and GMV in the MFG.L (r=0.296, P=0.025).


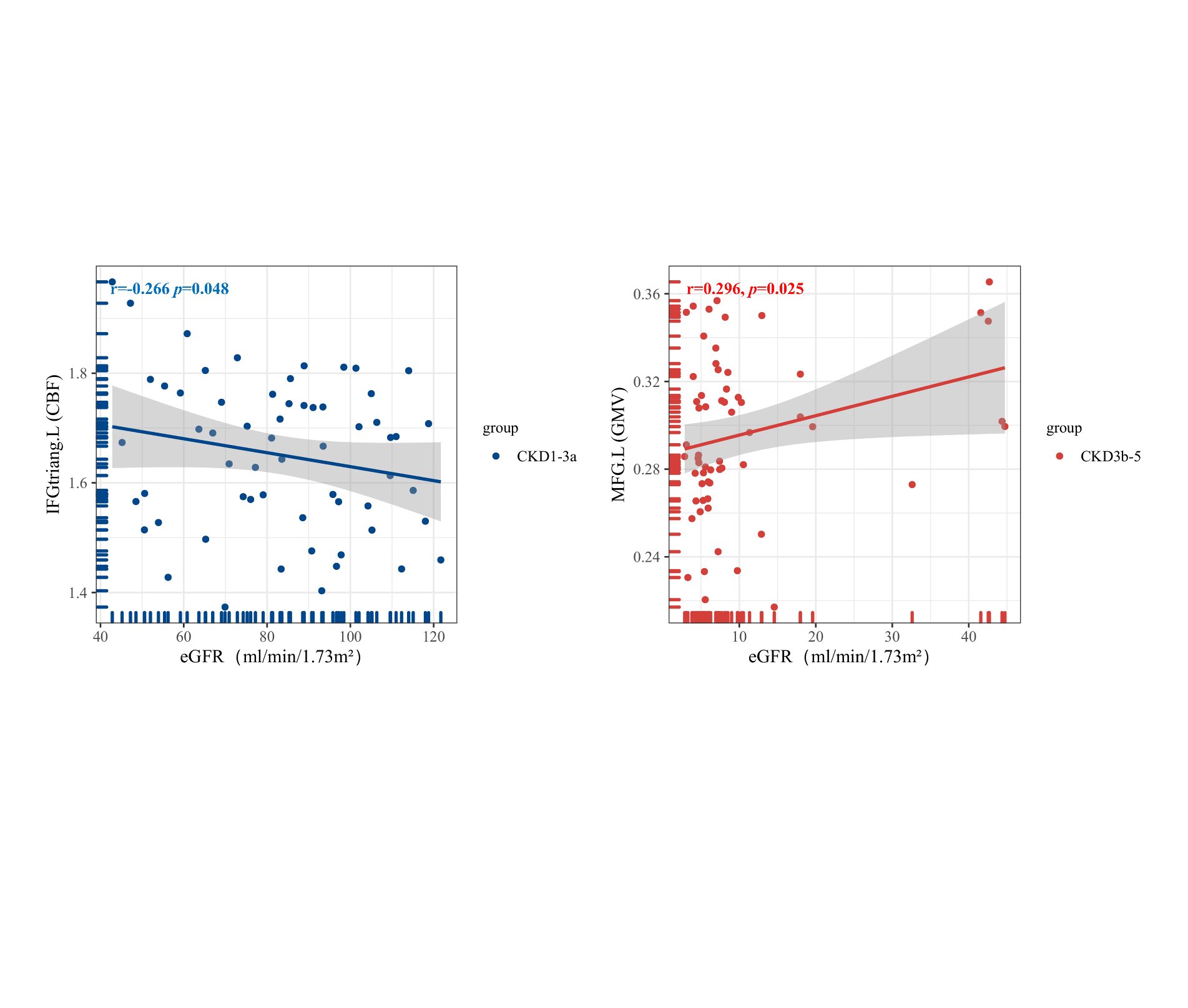


**Supplementary figure 1**. Correlation between the eGFR level and imaging parameters (CBF and GMV) in patients with different CKD stages. eGFR, estimated glomerular filtration rate; IFGtriang.L, left inferior frontal gyrus, triangular part; MFG.L, left middle frontal gyrus.
